# ADAM17 Is Critical for Multipolar Exit and Radial Migration of Neuronal Intermediate Progenitor Cells in Mice Cerebral Cortex

**DOI:** 10.1371/journal.pone.0065703

**Published:** 2013-06-03

**Authors:** Qingyu Li, Zhengyu Zhang, Zengmin Li, Mei Zhou, Bin Liu, Le Pan, Zhixing Ma, Yufang Zheng

**Affiliations:** School of Life Sciences, Fudan University, Shanghai, China; Zhejiang University School of Medicine, China

## Abstract

The radial migration of neuronal progenitor cells is critical for the development of cerebral cortex layers. They go through a critical step transforming from multipolar to bipolar before outward migration. A Disintegrin and Metalloprotease 17 (ADAM17) is a transmembrane protease which can process many substrates involved in cell-cell interaction, including Notch, ligands of EGFR, and some cell adhesion molecules. In this study, we used *in utero* electroporation to knock down or overexpress ADAM17 at embryonic day 14.5 (E14.5) in neuronal progenitor cells to examine the role of ADAM17 in cortical embryonic neurogenesis. Our results showed that the radial migration of ADAM17-knocked down cells were normal till E16.5 and reached the intermediate zone (IZ). Then most transfected cells stopped migration and stayed at the IZ to inner cortical plate (CP) layer at E18.5, and there was higher percentage of multipolar cells at IZ layer in the ADAM17-knocked down group compared to the cells in control group. Marker staining revealed that those ADAM17-knocked down cells differentiated normally from neural stem cells (NSCs) to neuronal intermediate progenitor cells (nIPCs) but did not differentiate into mature neurons. The migration and multipolar exit defects caused by ADAM17 knockdown could be partially rescued by over-expressing an shRNA resistant ADAM17, while overexpressing ADAM17 alone did not affect the radial migration. Taken together, our results showed for the first time that, ADAM17 is critical in regulating the multipolar-stage exit and radial migration of the nIPCs during telencephalon cortex development in mice.

## Introduction

The cerebral cortex has multiple-layer structure which is important for its functions. Such multiple-layer structure is controlled tightly during development process both temporally and spatially. During development, the neural stem cells in the subventricular zone (SVZ) differentiate into neuronal intermediate progenitor cells (nIPC). Then the nIPCs migrate along the radial glia fibers to the out layer of cortex and further differentiate into mature neurons. The later differentiated cells migrate longer distance to become the upper layer, such process has been cited as an “inside out” development process [1], [2]. During migration, the nIPCs have to undergo a multipolar stage before bipolar migration towards outer layer [3], [4]. The multipolar to bipolar transition process is a critical point of migration control and mistakes would cause server defects such as schizophrenia, epilepsy, autism, etc. [3], [4]. Such process can be affected by many molecules involved in regulating cell-cell interaction and cell skeleton [4], [5], [6], [7], [8], [9]. For example, knockdown Lissencephaly-1 Protein (LIS1) or Doublecortin (DCX) can cause multipolar cells accumulation in the VZ to IZ layers [5], [9]. What's more, recent studies showed that signaling pathways that are involved in regulating cell skeleton proteins can also affect multipolar to bipolar transition, such as reducing α-tubulin acetylation by knocking down its acetyltransferase Acetyltransferase mec-17 homolog (MEC-17) [6], or inhibiting phosphorylation of the microtubule regulator Superior cervical ganglion-10 protein (SCG10) by JNK knockout [7].

A Disintegrin and Metalloprotease 17 (ADAM17), also called Tumor Necrosis Factor alpha Converting Enzyme (TACE) was identified as the sheddase for Tumor Necrosis Factor alpha (TNFα) in 1997 [10], [11]. It is a type I transmembrane protease which belongs to the ADAMs family [12]. Besides TNFα, ADAM17 is also important for the releasing of many other transmembrane proteins that are important for cell-cell interaction and signaling, such as transforming growth factor alpha (TGFα), heparin-binding EGF-like growth factor (HB-EGF), Notch, amyloid precursor protein (APP), and some cell adhesion molecules [13], [14]. Some of those substrates have been reported to be involved in neural development. For example, proteolytic cleavage of the neural cell adhesion molecule (NCAM) by ADAM17 is involved in neurite outgrowth [15] and cleavage of neogenin by ADAM17 can desensitize cortical neurons to the repulsive guidance molecule [16]. Despite those *in vitro* studies, there is no report on *in vivo* function of ADAM17 during cortex development yet. On the other hand, ADAM10, a close family member of ADAM17, has been reported to be involved in cortex development as indicated by conditional knockout study [17]. Both ADAM10 and ADAM17 have been reported to be able to cleave Notch [18], [19], [20], which plays a critical role during cerebral development [21], [22]. However, the ADAM17 deficient mice have different phenotypes compare to Notch knockout mice. Instead, ADAM10 knockout mice have very similar phenotypes to Notch1 knockout mice [23]. Therefore, ADAM10, not ADAM17 is considered the physiological enzyme for Notch. Interestingly, recently van Tetering et al. and Bozkulak et al. reported simultaneously that ADAM10 is a ligand-dependent sheddase for Notch while ADAM17 is ligand-independent sheddase for Notch [18], [24]. This brings up new questions for ADAM17 function in Notch signaling pathway.

Besides Notch signaling, ADAM17 is also a critical enzyme for Epidermal Growth Factor Receptor (EGFR) signaling pathway [13], [14], [25], [26], [27]. ADAM17 is the major sheddase for the EGFR ligands TGFalpha and HB-EGF [28]. ADAM17 deficient mice die at birth due to cardiovascular and lung defects [29], [30], and the open-eye phenotype and skin development defects are similar to EGFR knockout and TGFalpha knockout mice [31], [32], indicating a critical role of ADAM17 in EGFR signaling pathway. However, those studies did not focus on cortex development. Although there is no report on *in vivo* function of ADAM17 during cortex development yet, it has been shown that EGFR level can affect progenitor cell fate in developing cortex [33] and EGFR signaling could affect neuronal cell migration demonstrated by using cortical slice [34] and EGFR knockout mice [35]. Therefore, it is very likely that ADAM17 is involved in cortex development as well.

In our study, we used *in utero* electroporation (IUE) method to either knock down ADAM17 expression by RNAi or overexpress ADAM17 in mice embryonic cortex. Our results revealed that ADAM17 is critical for controlling the radial migration of nIPCs in cortex development. After knocking down the expression of ADAM17 from E14.5 to E18.5 in mouse cortex, the outward migration of those neuronal intermediate progenitor cells was seriously slowed down with more cells in IZ stocked at multipolar stage.

## Results

### ADAM17 protein was expressed at cortical plate and intermediate zone during neurogenesis

**Figure 1:**
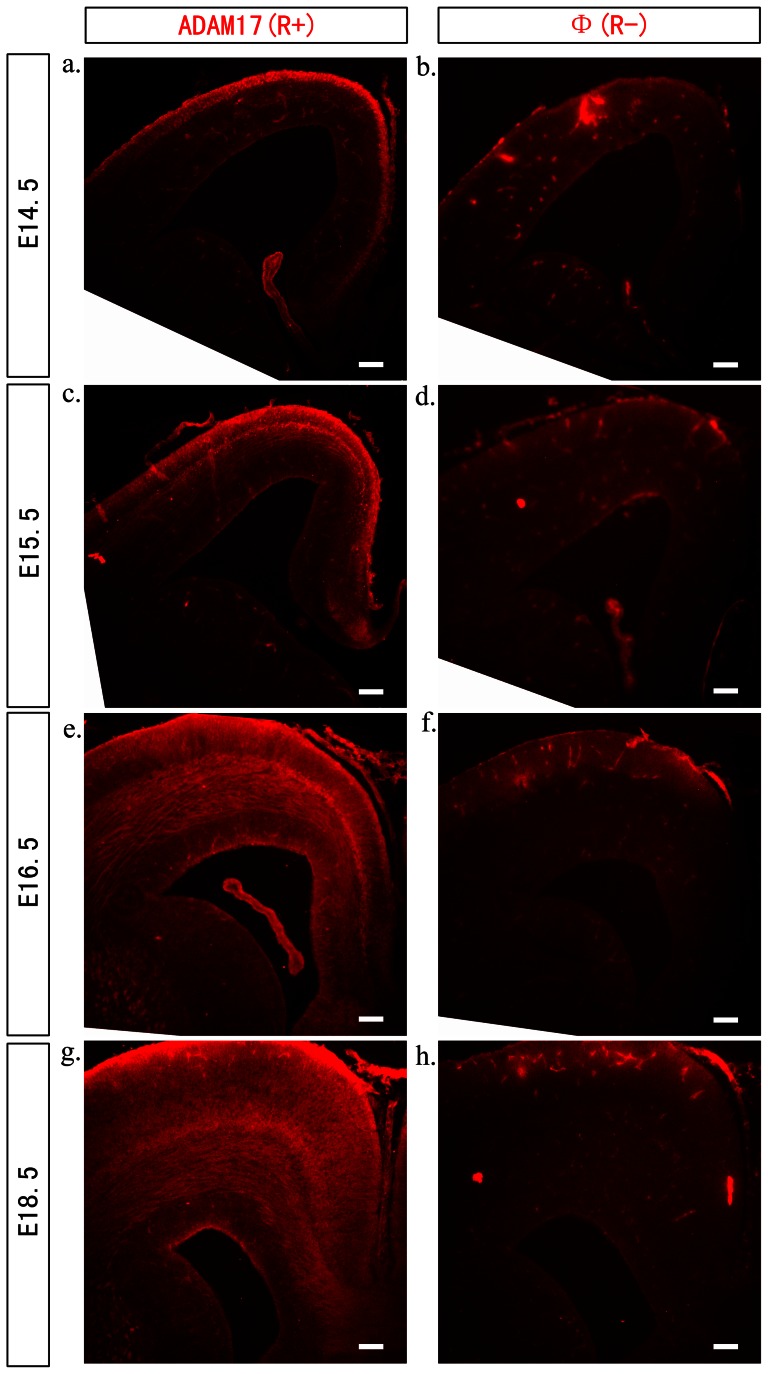
ADAM17 is expressed at CP and IZ layers during neurogenesis and migration period in mouse embryonic brain. Embryonic cerebral cortex from E14.5, E15.5, E16.5 and E18.5 mice were dissected out and sectioned at 10–15 µm for immunohistochemistry experiment with anti-ADAM17 antibody. ADAM17 is expressed at the margin zone at E14.5 (a) and gradually increased at CP and IZ layer at E15.5 (c), E16.5 (e), and E18.5 (g). Sections stained without primary antibody was included as negative control (b,d,f,h). The scale bar is 100 µm.

To investigate the role of ADAM17 during cortex development, we first determined its expression in developing cortex from E12.5 to E18.5 by westernblot (WB) and immunohisochemistry. WB on brain tissue sample showed that ADAM17 was expressed in developing mouse cortex with lower expression level at E12.5 and increased after E14.5 (Figure S1). Immunohistochemistry on brain tissue sections confirmed the expression of ADAM17 in developing cerebral cortex from E14.5 to E18.5 (Figure 1, panel a, c, e, g) and there was no staining on the control sections (Figure 1, panel b, d, f, h). At E14.5, ADAM17 expression was higher at the margin zone than the IZ to CP layer. Then its expression increased at the IZ to CP layer at E16.5 (Figure 1-e) and E18.5 (Figure 1-g) cortex. Such expressing pattern correlates with the peak neurogenesis and migration time during cortex development in mice. This indicates that ADAM17 may play a role during cortex development.

### ADAM17 deficiency leads to a radial migration defect

**Figure 2:**
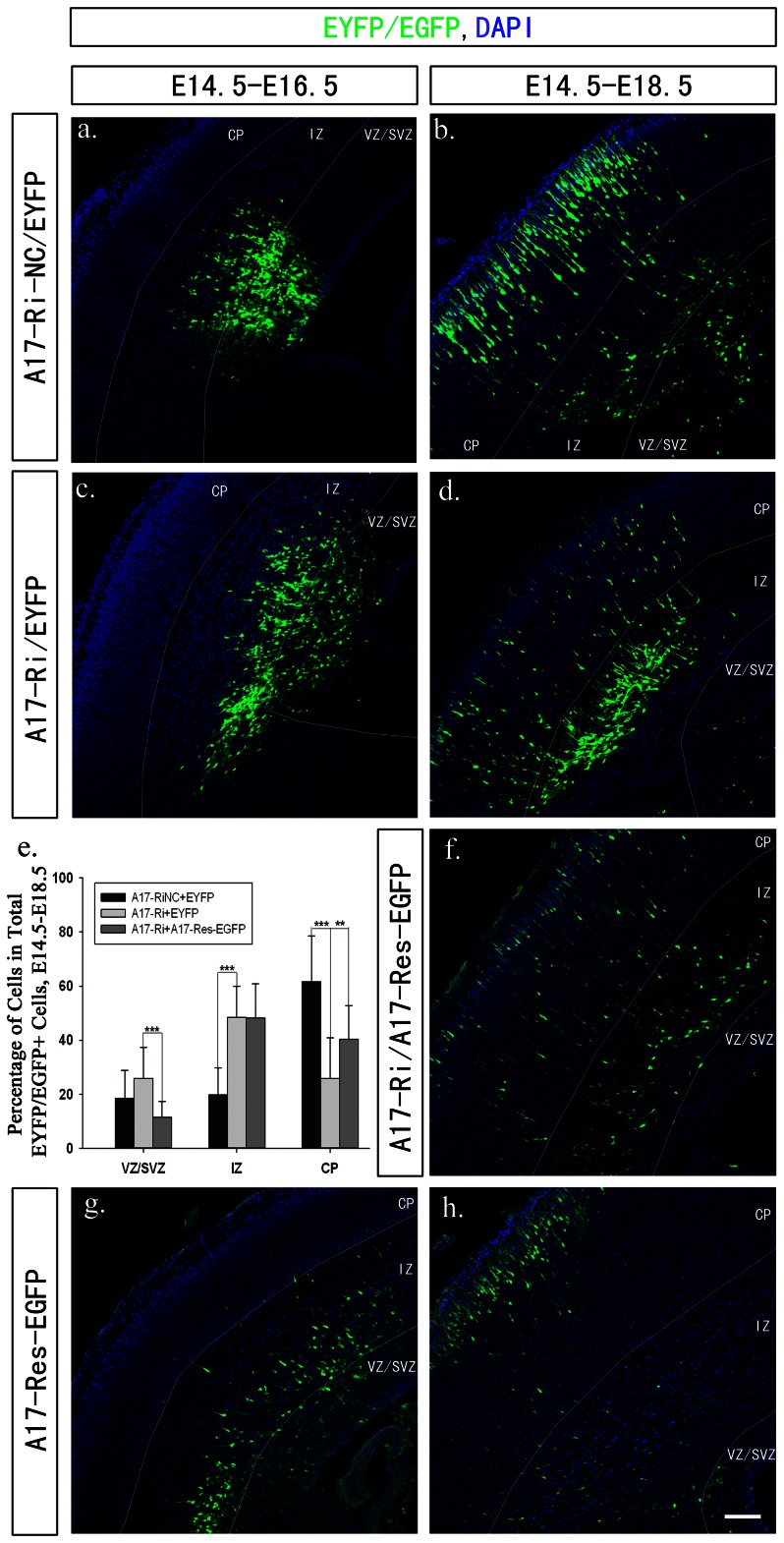
Knocking down ADAM17 expression could affect nIPC radial migration. Mouse embryos were electroprated at E14.5 with either shRNA constructs (A17-Ri or A17-Ri-NC) mixed with pEYFP or overexpressing construct A17-Res-EGFP respectively. Electroporated brains were collected at two different stages: E16.5 and E18.5, for immunohistochemistry experiments. All sections were cut at 20 µm. Electroporated cells were labeled by EYFP or EGFP. DAPI staining was used to indicate nuclei. The scale bar is 100 µm. At E18.5, most EYFP labeled neurons migrated to superficial CP layer in control group (b) while more cells stayed at IZ and inner CP layer in ADAM17 knockdown group (d). This radial migration defect caused by knocking down ADAM17 could be rescued by overexpressing shRNA resistant ADAM17 together with ADAM17 knockdown construct (f). Overexpressing A17-Res-EGFP did not affect the radial migration (h), and there was no obvious difference on the position and density of those electroprated cells at E16.5 (a, c, g). Quantitative analysis for cells position at different cortical regions at E18.5 was presented at (e) (*** P<0.001 and ** P<0.01).

To investigate the possible role of ADAM17 during cerebral cortex development, we then used IUE to knockdown ADAM17 at E14.5. The shRNA construct (A17-Ri) could efficiently knock down about 96% mouse ADAM17 (A17-HA) overexpressed in COS-7 cells (Figure S2). This shRNA construct was used in IUE to knockdown the endogenous ADAM17 in developing mouse cortex. A pEYFP construct was co-electroporated at 1∶6 ratio to the A17-Ri construct. The control group was co-transfected with a scramble shRNA construct and pEYFP construct. According to previous reports, EYFP is a reliable marker for co-electroporated cells and can be used for further quantitative analysis [5], [6], [7], [8], [36], [37], [38], [39]. The electroporated embryonic brain samples were collected at two different stages: E16.5 and E18.5, and processed for immunohistochemistry experiment and confocal microscope scanning. The results showed that knockdown ADAM17 in developing telencephalon caused a radial migration defect. There were 61.7% (61.7±16.8%) cells reached CP layer via radial migration in control group (Figure 2 b&e, Figure S3a–d) while only 25.7% (25.7±15.11%) cells reached CP layer in ADAM17-knocked down group at E18.5 (Figure 2 d&e, & Figure S3e–h), which was significantly lower than control group (P<0.001). There were more cells (48.4±11.5%) stayed in the IZ layer in the knockdown group (Figure 2d&e) while significantly less cells (19.8±9.9%, P<0.001) stayed in the IZ layer in the control group (Figure 2b&e). Such defect could be partially rescued by overexpressing a shRNA resistant ADAM17 (A17-Res-EGFP) together with A17-Ri construct at 1∶6 ratios. A17-Res-EGFP was generated by synonymous mutation at the shRNA targeting sequence to create a shRNA resistant ADAM17 without changing the amino acids. As shown in figure 2f, the migration defect caused by ADAM17 specific shRNA was partially restored by overexpressing A17-Res-EGFP as about 40.2% (40.2±12.5%) cells reached the CP layer, which was significantly more than the knockdown group (P<0.01) (Figure 2f&e, Figure S3 i–l). On the other hand, cells overexpressing A17-res-EGFP alone did not have a significant effect on radial migration as most EGFP positive cells also reached the superficial CP layer (Figure 2h). We also checked the early migration at E16.5, which is two days after IUE. We did not observe any significant change at E16.5 in all three groups as all EYFP/EGFP positive cells were located at VZ/SVZ to inner IZ layer (Figure 2 a, c, g).

### Knocking down ADAM17 expression level affected the radial glia fiber alignment and the multipolar to bipolar transformation of neuronal progenitor cells

**Figure 3:**
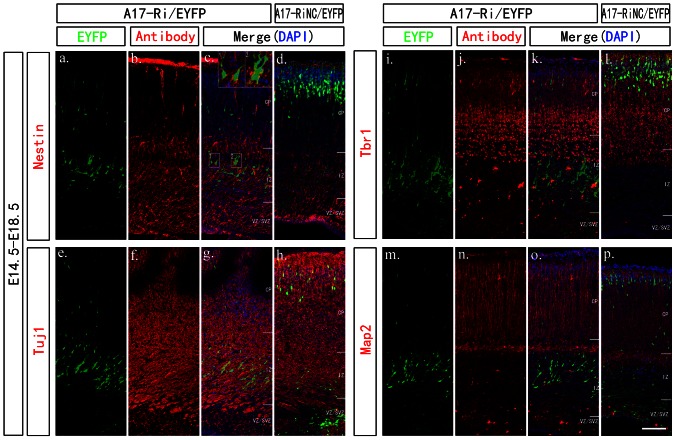
ADAM17 deficiency affected NSCs differentiation and those cells stopped at nIPCs stage as indicated by markers staining. Mouse cortex electroporated at E14.5 with A17-Ri/EYFP were dissected out at E18.5. All sections were cut at 20 mm and processed for IF with antibodies against one of those following markers: Nestin (b), Tuj1 (f), Tbr1 (j) and Map2 n). DAPI staining was used to indicate nuclei and merged images were presented in c-d, g-h, k-l, o-p. The scale bar is 100 mm. Those ADAM17 deficient cells were stained positive with Nestin (c) and Tuj1 (g), but not with Tbr1 (k) or Map2 (o).

To investigate whether the radial migration defect in ADAM17 knock down group would affect the cortex structure, we next performed immunofluoresent experiment with Nestin (a neural stem cell marker), Tuj1 (β-III-tubulin, a neuronal marker), Tbr1 (an early neuron marker), Map2 (a mature neuron marker), and L1-CAM (neuronal axon tract marker) on electroporated brain sections. As the electroporated cells were only a small population, the overall cortical layer structures were not affected in either group and there was no obvious change on Nestin, Tuj1, Tbr1, and L1-CAM staining (Figure 2 & S3). Closer examination on ADAM17 knockdown group showed that those cells stocked at IZ layer at E18.5 were positive for Nestin and Tuj1 staining (Figure 3 a–c & d–f) but negative for Tbr1 or Map2 staining (Figure 3 g–i & j–l). It suggested that those cells differentiated normally from neural stem cell at VZ-SVZ layer into neuronal intermediate progenitor cells (nIPCs) at IZ layer, but did not differentiate into neuron cells.

**Figure 4:**
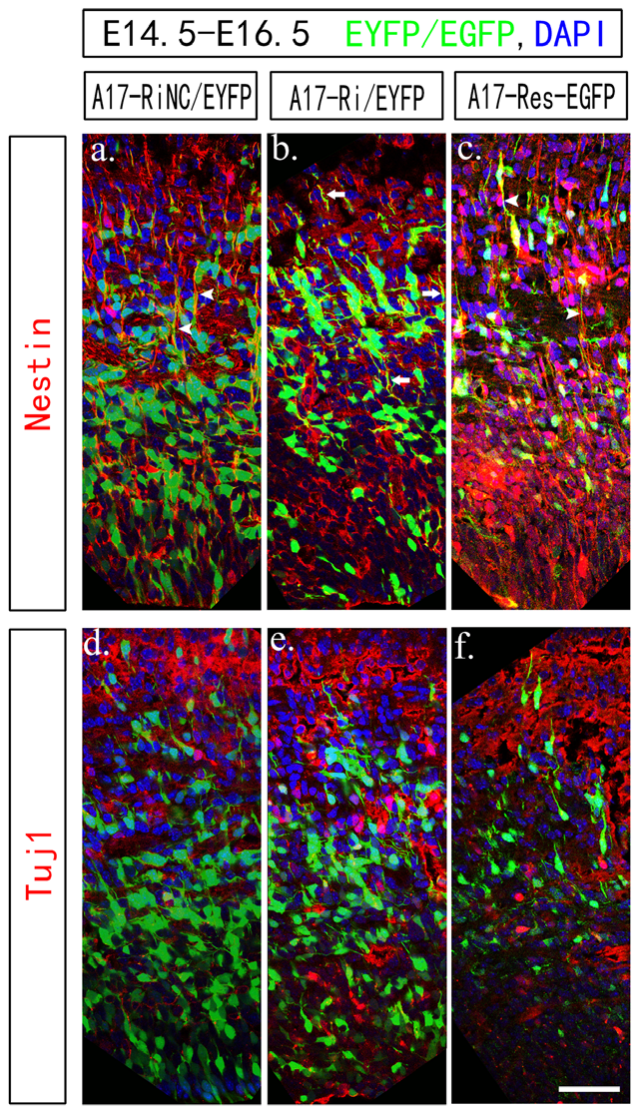
Knocking down ADAM17 expression affected the radial glia fiber at E16.5 in cerebral cortex. Mouse embryos were electroporated at E14.5 and dissected at E16.5 for confocal imaging with Nestin or Tuj1 staining. All sections were cut at 20 µm. Each group was shown as the label on top. DAPI staining was used to indicate nuclei. All scale bars are 100 µm. All images presented the VZ/SVZ to IZ layers with the superficial layer on top. The radial glia fibers in the control group and overexpressing group were smoother and better aligned as indicated by the white arrow heads in (a, c). The radial glia fibers in the ADAM17 knockdown group appeared with more branches and not as well aligned, indicated by white arrows in (b).

As the radial glia cells, which are neural stem cells [40], also serve as the scaffold for nIPCs radial migration, a disruption on radial glia fibers could also affect outward migration. We then examined carefully on those radial glia fibers by Nestin staining at E16.5. As shown in figure 4, most EYFP/EGFP positive cells were Tuj1 negative (Figure 4 d–f) and Nestin positive (Figure 4 a–c) at E16.5, suggesting they were indeed neural stem cells. However, the radial glia scaffold were not as well aligned in knockdown group and those cells had processes with more branches (Figure 4b, white arrow heads) compared to the long and aligned fibers in control group (Figure 4a, white arrows). Overexpressing A17-Res-EGFP did not affect the radial glia fiber alignment (Figure 4c).

**Figure 5:**
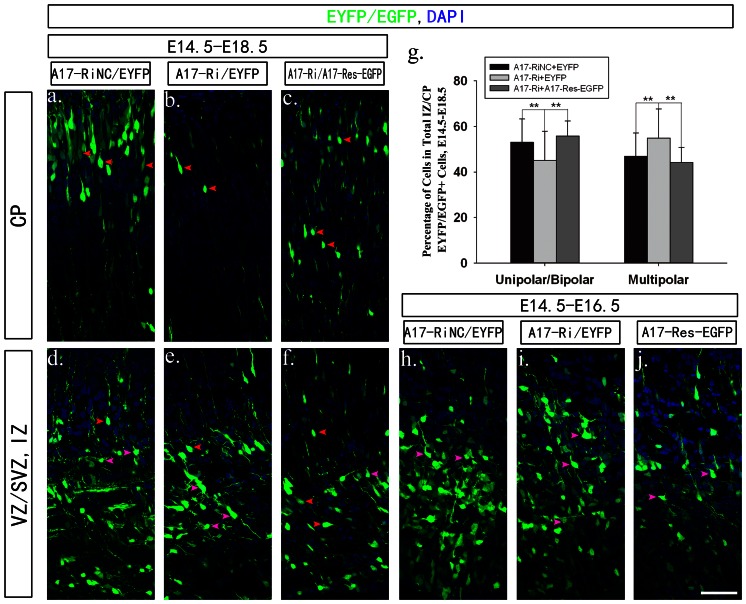
Knocking down ADAM17 expression affected the multipolar to bipolar transition in cerebral cortex. Mouse embryos were electroporated at E14.5 and dissected at E16.5 and E18.5 for confocal imaging. All sections were cut at 20 µm. Each group was shown as the labels. DAPI staining was used to indicate nuclei. All scale bars are 100 µm. The CP and VZ/SVZ layers were presented seperated. The CP layers of E18.5 were presented in (a–c) and the VZ/SVZ layers of E18.5 were presented in (d–f). The VZ/SVZ layers of E16.5 were presented in (h–j). There were more multipolar stage cells in the ADAM17 knockdown group at E18.5 indicated by purple arrow heads. The statistic analysis was presented in (g, ** P<0.01).

Next, we looked carefully at the morphology of those transfected cells at both E16.5 and E18.5. At early migration time (E16.5), cells had just reached the IZ layer and all three groups had similar amounts of multipolar cells (Figure 5 h–j). On the other hand, although those cells reached CP layer in all groups had uniform bipolar morphology at E18.5 (Figure 5 a–c), there were more cells staying at multipolar stage in IZ layer in the ADAM17 knockdown group compared to the control group (Figure 5 d&e). Statistic analysis showed the difference between two groups was about 8% (46.9±10.2%% in control group and 54.9±12.8% in knockdown group) and significant (P<0.01) (Figure 5g). Such defect could also be rescued by overexpressing A17-Res-EGFP (Figure 5 f&g, 44.2±6.6% multipolar, P<0.01 compared to knockdown group). Therefore, ADAM17 is probably critical for both radial glia fibers formation and multipolar to bipolar transition of nIPCs in developing cerebral cortex.

## Discussion

In our study, we used ADAM17 specific shRNA and IUE to knockdown its expression in mice embryonic cortex. Our results revealed that ADAM17 is critical for the radial glia fiber formation and the nIPCs multipolar exit and migration during mouse cortex development. To our knowledge, this is the first report on the *in vivo* function of ADAM17 in cortex development.

Although both ADAM17 and ADAM10 have been reported to be able to cleave Notch receptor [18], [19], [20], the ADAM17 deficient mice had different phenotypes compared to Notch knockout mice [29], [30], [31], [32]. In our experiments, after knocking down ADAM17 expression in the NSCs, the NSCs population was not affected and they could still differentiate into normal nIPCs but not mature neurons as revealed by marker staining (Figure 3). Such phenotype is very different from ADAM10 conditional knockout mice as the NSCs were significantly affected in those mice [17]. Therefore, it is unlikely that knocking down ADAM17 expression affected the Notch signaling pathway under our condition. Such results is in consistent with the reports that ADAM10 is more or less the physiological sheddase for Notch receptor [18], [24].

The next question will be what the target of ADAM17 is during cortex development. We observed that both migration and differentiation of nIPCs were blocked by ADAM17 knockdown. There are several substrates of ADAM17 have been reported to be able to affect nIPCs migration and/or differentiation, such as HB-EGF [34], Neuregulin 1 (NRG1) [41], L1-CAM [42], etc. Neuregulin1 is a member of EGFR ligand family. It has been reported that NRG1 could affect neuron migration on cortical slice [41], [43], [44], however, its mRNA expression in developing telecephalon is located at the VZ but not IZ to CP layer [45], [46]. HB-EGF is another EGFR ligand, which has been reported to express at inner CP to IZ layer at E14–15 in mice cortex [34]. Previous studies showed that when HB-EGF is specifically knockout in the ventral forebrain, the mice showed abnormal psychiatric behaviors and decreased spine density in neurons of the prefrontal cortex [47]. However, the cortex development has not been carefully examined in those mice and further investigation will be needed. Besides EGFR ligands, cell adhesion molecule L1-CAM, an ADAM17 substrate highly expressed at IZ [48], has also been shown to be able to affect neuron migration in explants assay [42]. It is possible that ADAM17's cleavage of L1 is critical for nIPCs to pass IZ. It is very possible that multiple ADAM17 substrates are involved in radial glia fiber formation and nIPC migration. Further experiments on conditional knockout mice with different promoters will very beneficial to investigate the molecular mechanisms of ADAM17 in cortex development.

Our results showed that ADAM17 knockdown affected the radial migration of nIPCs and the alignment radial glia fibers at E16.5. Such alignment defect could contribute to the migration defect as normal radial glia fibers is necessary for nIPCs migration [49], however to what degree and through which mechanism are unclear and need further investigation. Our results also indicated that E16.5 to E18.5 time period is critical for the transition of multipolar nIPC to biopolar early neurons during telencephalon development. This multipolar stage is extremely sensitive and slight disruption can cause neuronal migration defects [3], [50]. Recently, two studies showed that phosphorylation of microtubule could regulate the multipolar-stage exit and radial migration [7], [51]. It is possible that ADAM17 could affect the phosphorylation of cytoskeleton via EGFR signaling pathways which in turn affect the multipolar-stage exit and radial migration.

The expression of ADAM17 must be tightly controlled during cortex development to achieve proper timing. This could be achieved by either transcriptional regulation or post-transcriptional regulation. There is no such study yet in cortex development, but some information about regulation of ADAM17 expression are available in cancer and inflammation fields. For transcriptional regulation, it has been reported that hypoxia could induce ADAM17 expression and such induction can be mediated through hypoxia-inducible factor-1 (HIF-1) [52] and SP1 [53]. The substrate of ADAM17, TNFα, could also induce ADAM17 upregulation through NFκB pathway [52], [54]; while Ron receptor could down-regulate ADAM17 expression by inhibiting NFκB activity [55]. For post-transcriptional regulation, it has been shown that ADAM17 expression level could be post-transcriptionally upregulated by EGFR activation in breast tumor [56] or by phospholipase A(2) treatment in human neuroblastoma SK-N-SH cells [57]. However, as the mechanisms for the regulations on ADAM17 are scare, large scale analysis on what factors are responsible for ADAM17 expression during cortex development will be needed in the future.

## Materials and Methods

### Ethics Statement

All C57BL/6 mice were purchased from Shanghai SLAC laboratory animal Inc. This study was carried out in strict accordance with the recommendations in the Guide for the Care and Use of Laboratory Animals of Fudan University. The protocol was approved by the Committee on the Ethics of Animal Experiments of Fudan University, Shanghai, People's Republic of China. All surgery was performed under sodium pentobarbital anesthesia (intraperitoneal injection of 5 mg/ml Pentobarbitalum Natricum at 12 µg/g body weight) and all efforts were made to minimize suffering.

### Plasmids

Mouse ADAM17 expression vector pcDNA3-ADAM17-HA was kindly provided by Dr. Carl P. Blobel. ADAM17 RNAi constructs were purchased from GenePharma Inc. To generate mouse ADAM17 shRNA, the fellowing hairpin was cloned into pGPH1/GFP/Neo vector (GenePharma): 5′-CACCGGATTAGCTTACGTTGGTTCTTTCAAGAGAAGAACCAACGTAAGCTAATCCTTTTTTG-3′, named as A17-Ri. A scramble sequence 5′-CACCGTTCTCCGAACGTGTCACGTCAAGAGATTACGTGACACGTTCGGAGAATTTTTTG-3′ was cloned into the same vector served as control A17-Ri-NC (GenePharma).

To generate shRNA resistant ADAM17, the targeting site of shRNA was mutated into 5′-GGCCTGGCCTATGTGGGCAGC-3′ from 5′-GGATTAGCTTACGTTGGTTCT-3′ (1045 bp–1065 bp on cDNA sequence) by site-mutagenesis kit (Takara). Then the shRNA resistant ADAM17 was amplified out by PCR with the following primers (Forward 5′-aaaagatctatgaggcggcgtctcct-3′ and Reverse 5′-aaaaccggtttaagcataatctgga-3′) and cloned into pCAG-IRES-EGFP vector between Bgl II and XmaI sites for IUE experiments.

### Cell Culture and Western Blot

COS-7 cells were obtained from the Cell Bank at the Chinese Academy of Sciences for this study. COS-7 cells were grown in Dulbecco's modified Eagle's medium supplemented with 10% fetal bovine serum, 2 mM glutamine, and 1% penicillin/streptomycin and cotransfected with mouse ADAM17 expression plasmids (pcDNA3-A17-HA or pcDNA3-A17-Res-HA) and ADAM17 shRNA constructs (A17-Ri) with Fugene6 (Roche) according to the manufacturer's protocol. Forty-eight hours after transfection, cells were washed with PBS, then lysed in 100 µl of RIPA buffer (50 mM Tris-HCl pH7.4, 150 mM NaCl, 1% TritonX-100, 1% Sodium Deoxycholate, 0.1% SDS) plus 5 mM 1,10-phenanthroline and protease cocktail (Thermo 78410) per 35 mm dish. Lysates were cleared by centrifugation at 13,000 rpm for 30 min. The supernatants were subjected to western blot. In brief, proteins samples mixed with sample loading buffer were loaded and separated by SDS-PAGE and then electrically blotted to a polyvinylidene difluoride (PVDF) membrane (Amersham). After overnight incubation in blocking buffer (10% non-fat dry milk in TBST buffer 50 mM Tris-HCl pH 7.4, 150 mM NaCl, 0.1% Tween20) at 4°C, immunoblots were incubated with anti-HA antibody (Beyotime) in TBST buffer overnight at 4°C followed by HRP-conjugated goat anti-mouse secondary antibody (Beyotime) for 1 hrs at room temperature. Blots were then washed with TBST buffer and incubated with enhanced chemiluminescence (ECL, Millipore) and detected by Kodak Film. Density were detected with software Quantity One and GAPDH was used as loading control.

Mouse brains were dissected out at different embryonic stages and then lysed in RIPA buffer (Beyotime) plus 5 mM 1,10-phenanthroline and protease cocktail (Thermo 78410). Protein concentrations were quantified by using BCA kit (Beyotime). Then 50 µg proteins from each stage (E12.5, E14.5, E16.5, and E18.5) were subjected to Western analysis with goat anti-ADAM17 antibody ab13535 (Abcam) and HRP conjugated donkey anti-goat antibody (Shanghai Kangchen Inc.) as described above.

### In utero electroporation

In utero electroporation was performed as previously described [5], [6], [7], [8], [36], [37], [38], [39]. Timed-pregnant female mice (14.5 days) were purchased from SLAC laboratory animals Inc., Shanghai, China. Mice were anaesthetized by intraperitoneal injection of 5 mg/ml Pelltobarbitalum Natricum at 12 µg/g body weight. Uteri were carefully exposed through a 1.5-cm incision in the ventral peritoneum and placed on humidified gauze pads. DNA prepared using Qiagen Endo Free plasmid purification kit mixed with 0.05% Fast Green (Sigma) was injected through the uterine wall into the telencephalic vesicle using pulled borosilicate glass needles (Sutter Instrument). The DNA mix was prepared with the shRNA plasmid A17-Ri or A17-Ri-NC to pEYFP plasmid at 6∶1 ratio (in µg). For rescue experiments, the ratio of A17-res-HA-EGFP to A17-Ri was 1∶6 (in µg). Five electrical pulses were applied at 30 V (50 ms duration, 1 Hz frequency) across the uterine wall using 5-mm platinum tweezers electrodes and an ECM830 BTX square wave electroporator (BTX, Gentronic, Inc.). The uterine horns were then carefully placed back into the abdominal cavity, and the abdomen wall and skin were sutured separately. Mice were placed at warm place until it woke up and then moved back to an individual cage for recovery. Two days (E16.5) or four days (E18.5) after the surgery, pregnant mice were sacrificed by neck dislocation, and embryos were processed for tissue analyses.

### Immunohistochemistry and microscopy

Embryonic brains were fixed in 4% paraformaldehyde in PBS overnight and placed in 15% sucrose/PBS for 12 h and then 30% sucrose/PBS for 24 h at 4°C. Brains were then embedded in OCT (optimum cutting temperature) mounting medium and froze before sectioning at 10 or 20 µm using a cryostat (Leica CM1900). The following primary antibodies were used: goat anti-ADAM17 (Abcam, ab13535); rabbit anti-Tbr1 (Abcam, 31940), anti-Tbr-2 (Abcam, ab23345), anti-GFP (Beyotime); mouse anti-Nestin (Abcam, ab6142), anti-Tuj1 (class III beta-tubulin, Abcam, ab78078), anti-Map2 (Abcam, ab11268); and rat anti-L1-CAM (Millipore, MAB5272) antibodies. Sections were then incubated with according secondary Cy3-labeled goat antibody (anti-rabbit, anti-mouse, or anti-rat, all purchased from Beyotime), Cy5-labeled donkey anti-goat secondary antibody (Abcam, ab6566), or FITC-labeled goat anti-rabbit secondary antibody (Beyotime). Negative control was detected with secondary antibody alone. Fluorescent images were taken by using Olympus IX71 microscope and Zeiss LSM700 confocal microscope.

### Cell Counting and Statistics

Nuclear cell staining with 4′,6-diamidino-2-phenylindole (DAPI) was used to define different sub-regions of the cerebral cortex (SVZ/VZ, IZ, and cortical plate [CP] based on cell density as previously described [38]. In all experiments, slices from at least 3 independent experiments were processed. The proportion of EYFP or EGFP positive cells in each region was counted. Sections were analyzed for each condition from at least 3 embryos from 2–3 parallel experiments. Multipolar and bipolar cells were distinguished on the basis of morphology. Cells with more than two extensions were scored as multipolar. Results are indicated as mean ± standard error of the mean (SEM). Statistical analysis was performed by using student T-test in Excel with P<0.05 as significant difference.

## Supporting Information

Figure S1 ADAM17 expression in mice embryonic brain tissue by WB. Embryonic cerebral cortex from E12.5, E14.5, E16.5, and E18.5 mouse embryos were dissected out and lysed. 50 µg protein each lane were loaded onto the gel to process for WB. GAPDH were used as loading control.
(TIF)

Figure S2 The ADAM17 specific shRNA construct could knockdown ADAM17 expression efficiently on COS-7 cells. Cells were co-transfected with mouse A17-HA or A17-Res-HA constructs and shRNA constructs at 1∶1 ratio as indicated on the top of panel a. Two days later, cells were processed for WB. Density were detected with software Quantity One and GAPDH was used as loading control. A representative WB image was shown in (a) and GAPDH was used as loading control. The statistic analysis was shown in (b) and the A17-RiNC+A17-HA group was calculated as 100%.
(TIF)

Figure S3 Marker staining on electroprated brain sections for control, knockdown and rescue groups. Mouse cortex electroporated at E14.5 were dissected out at E18.5. All sections were cut at 20 µm and processed for IF with antibodies against one of those following markers: Nestin (a,e,i), Tuj1 (b,f,j), Tbr1 (c,g,k) and L1-CAM (d,h,l). DAPI staining was used to indicate nuclei. The scale bar is 100 µm.
(TIF)
