# Supplementary figures and images for: ADAM17 Is Critical for Multipolar Exit and Radial Migration of Neuronal Intermediate Progenitor Cells in Mice Cerebral Cortex
Source: PLoS One. 2013 Jun 3;8(6):e65703. doi: 10.1371/journal.pone.0065703 (PMC3670835; doi:10.1371/journal.pone.0065703)

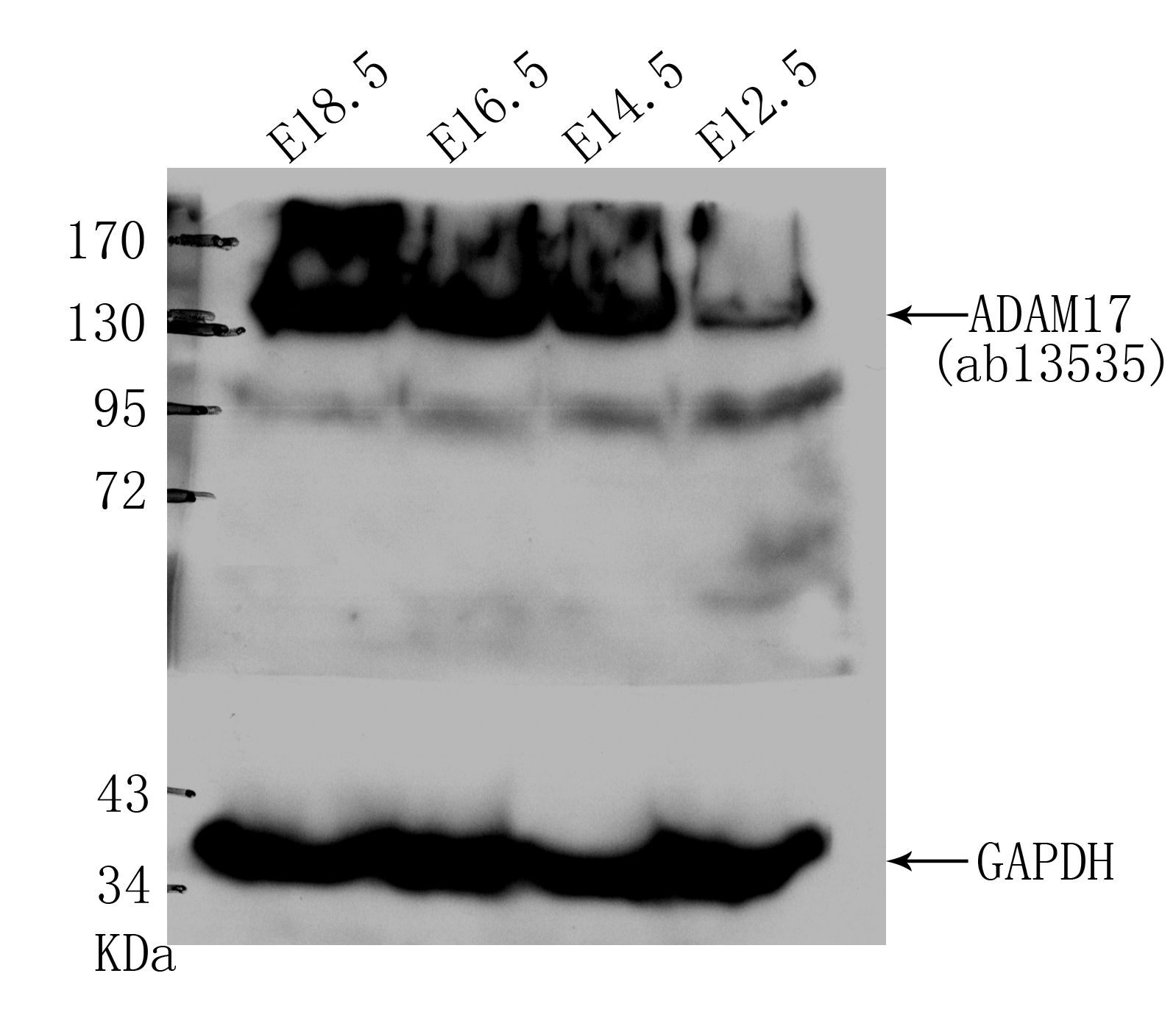

Supplement: Figure S1 — ADAM17 expression in mice embryonic brain tissue by WB. Embryonic cerebral cortex from E12.5, E14.5, E16.5, and E18.5 mouse embryos were dissected out and lysed. 50 µg protein each lane were loaded onto the gel to process for WB. GAPDH were used as loading control. (TIF) [file pone.0065703.s001.tif]

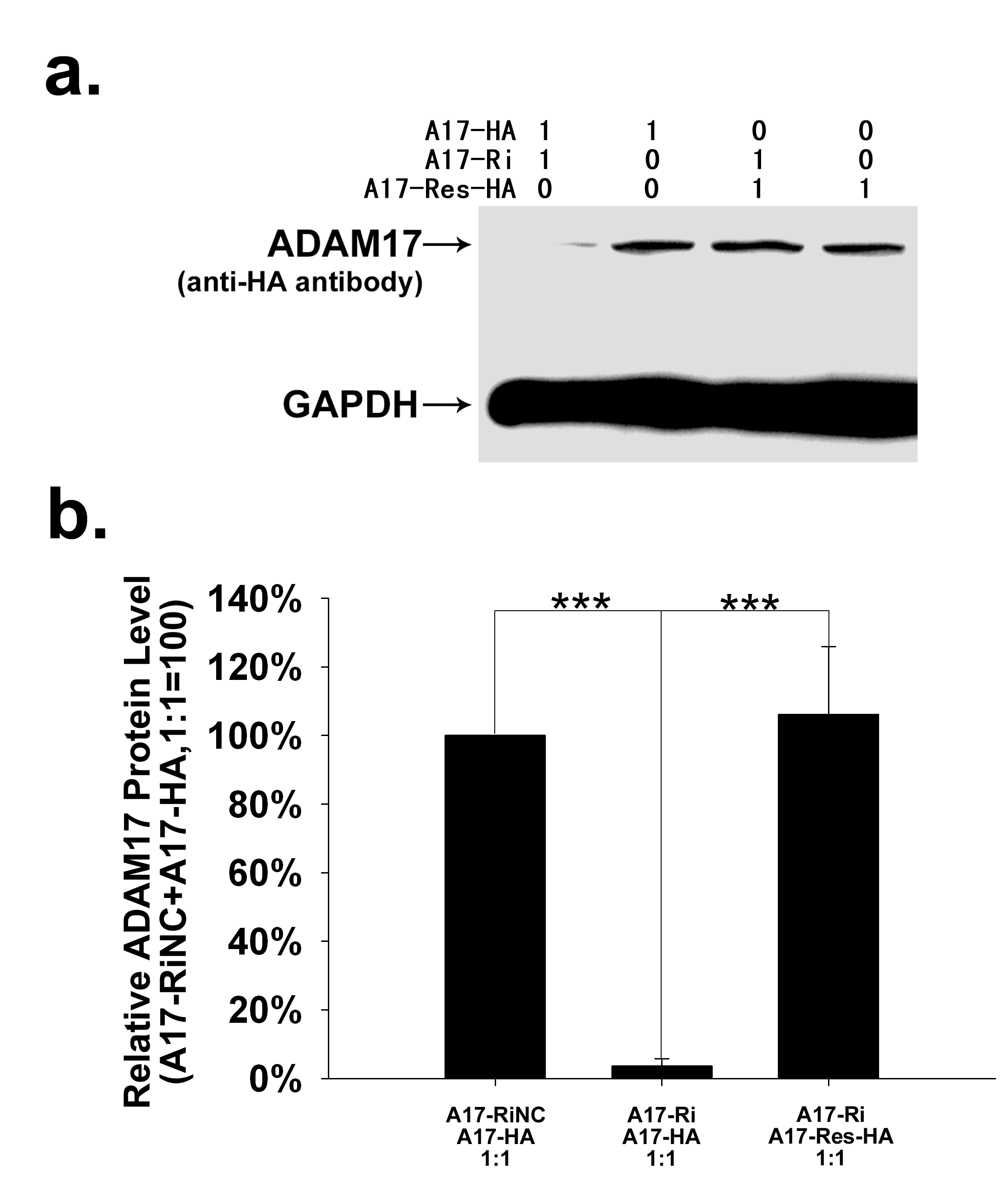

Supplement: Figure S2 — The ADAM17 specific shRNA construct could knockdown ADAM17 expression efficiently on COS-7 cells. Cells were co-transfected with mouse A17-HA or A17-Res-HA constructs and shRNA constructs at 1∶1 ratio as indicated on the top of panel a. Two days later, cells were processed for WB. Density were detected with software Quantity One and GAPDH was used as loading control. A representative WB image was shown in (a) and GAPDH was used as loading control. The statistic analysis was shown in (b) and the A17-RiNC+A17-HA group was calculated as 100%. (TIF) [file pone.0065703.s002.tif]

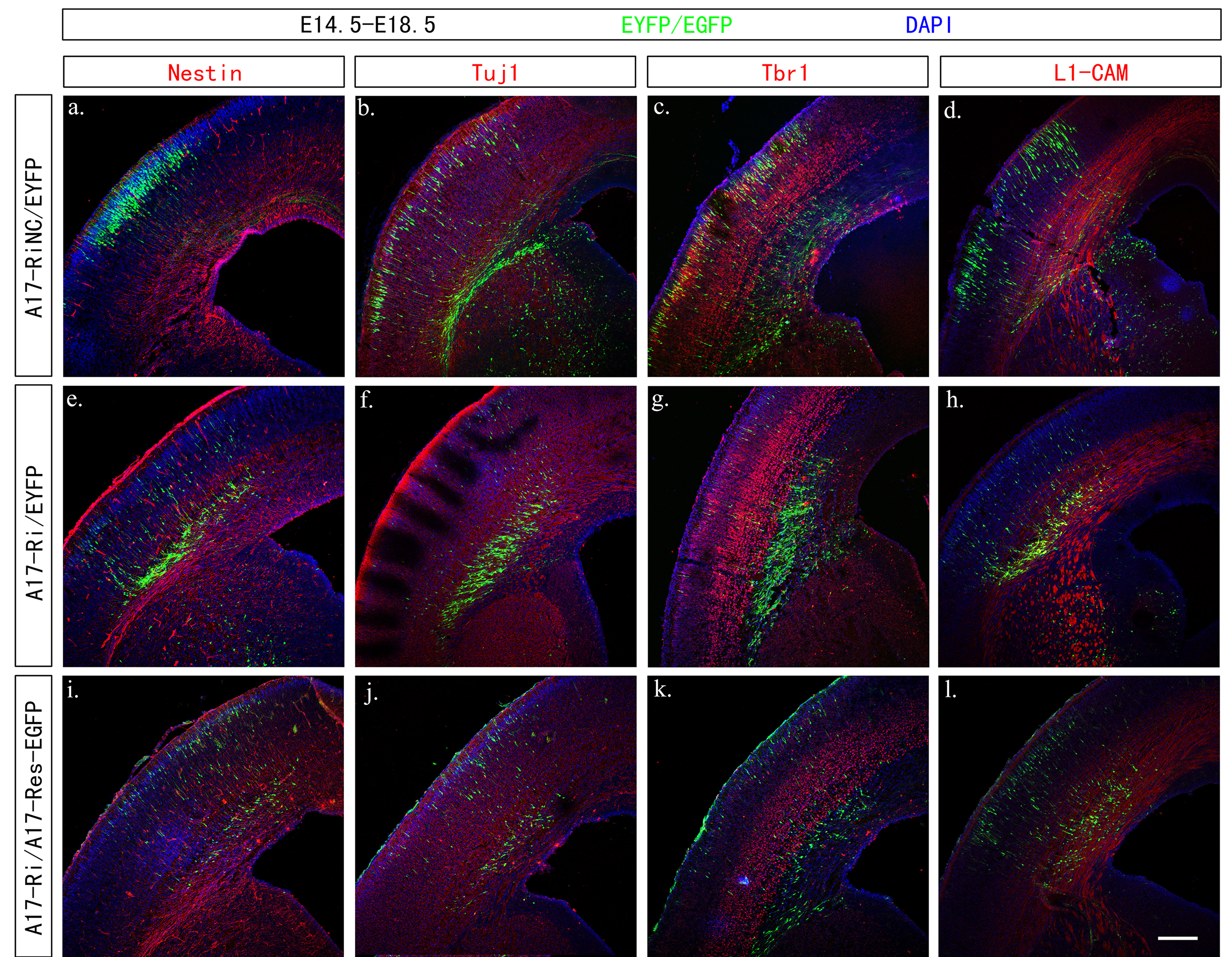

Supplement: Figure S3 — Marker staining on electroprated brain sections for control, knockdown and rescue groups. Mouse cortex electroporated at E14.5 were dissected out at E18.5. All sections were cut at 20 µm and processed for IF with antibodies against one of those following markers: Nestin (a,e,i), Tuj1 (b,f,j), Tbr1 (c,g,k) and L1-CAM (d,h,l). DAPI staining was used to indicate nuclei. The scale bar is 100 µm. (TIF) [file pone.0065703.s003.tif]
